# Cascaded gain-switching in the mid-infrared region

**DOI:** 10.1038/s41598-017-17305-1

**Published:** 2017-12-04

**Authors:** Hongyu Luo, Jianfeng Li, Chen Zhu, Xue Lai, Yongchen Hai, Yong Liu

**Affiliations:** 1 State Key Laboratory of Electronic Thin Films and Integrated Devices, School of Optoelectronic Information, University of Electronic Science and Technology of China (UESTC), Chengdu, 610054 China; 2 Science and Technology on Solid-State Laser Laboratory, 11th Research Institute of China Electronics Technology Group Corporation, Beijing, 100015 China

## Abstract

In this report, we demonstrate mid-infrared dual-waveband (i.e., ~3 μm and ~2 μm) pulses from a cascaded gain-switched Ho^3+^-doped ZBLAN fiber laser by the use of hybrid pumping of 1150 nm CW and pulse LDs for the first time. Stable ~3 μm gain-switched pulses with the maximum output power 262.14 mW and shortest pulse duration of 0.824 μs were first gained at the repetition rate of 80 kHz and wavelength of 2928.5 nm. Then stable ~2 μm gain-switched pulses at 2068 nm were achieved at a switchable repetition rate between 40 kHz and 80 kHz. The maximum output power and shortest pulse duration were 75.23 mW and 0.787 μs, respectively (not simultaneously). Between them, there is a power-dependent μs-order time delay. This dual-waveband laser source has great potential in laser surgery, material processing.

## Introduction

Pulsed laser sources in the mid-infrared region have attracted large amount of attention as a result of their widespread applications in surgery, remote sensing, defense, industrial fields etc., in which optical fiber based platform has the outstanding merits of great beam quality, good heat dissipation, high optical-to-optical conversion efficiency and compact structure thus been paid more attention^1^. In the past decade, its wavelength has been extended to ~3 μm region from the well concerned ~2 μm region, motivated by urgent practical demands in laser surgery^2,3^ and material processing^4^ owing to the strong absorption of OH bonds around 2.94 μm^5^. Mode-locking, as a feasible way to achieve ps- and fs-order pulses^6–8^, has been successfully applied in Er^3+^- and Ho^3+^-doped ZBLAN fiber lasers emitting at ~3 μm based on either material saturable absorbers or NPR structure^5^. The shortest pulse duration and largest peak power have been down and up to 180 fs and of 37 kW, respectively^9^. To gain ns- and μs-order pulses, Q-switching resorting to intra-cavity modulators is an alternative technique approach, and so far various modulators (e.g., AOM, SESAM, Fe^2+^:ZnSe, 2D materials) have been introduced into Er^3+^- and Ho^3+^- doped ZBLAN fiber lasers to generate Q-switched pulses at 3 μm waveband^5^. Using AOM based active Q-switching, the shortest pulse duration of 78 ns^10^, largest average output power of 12 W^11^ and pulse energy of 0.15 mJ^12^ have been achieved, respectively (not simultaneously). Although compact and robust passive Q-switching is much more preferred, its output cannot reach the same level as active Q-switching currently. By contrast, dual-waveband pulsed fiber lasers combining ~3 μm with ~2 μm would undoubtedly offer more virtues under some conditions where the waveband is optionally required (e.g., multi-material processing^4,13^) or both are simultaneously needed (e.g., efficient and safe tissue ablation^14–16^). Fortunately, the cascaded transitions (i.e., ^5^I_6_ → ^5^I_7_ and ^5^I_7_ → ^5^I_8_) of Ho^3+^-doped ZBLAN fiber are just located at the ~3 μm and ~2 μm bands^17^ which provide the opportunity of dual-waveband pulsing. Recently, we adopted AOM/SESAM as a Q switcher to realize ~3 μm and ~2 μm dual-waveband pulses using Ho^3+^-doped ZBLAN fiber^18,19^.

Compared to Q-switching, gain-switching, periodically modulating laser gain by pulse pumping, owns two remarkable merits preferred by applications. First, it doesn’t need any intra-cavity modulators which makes the system simple and compact, and prevents the limitation factor of pulse power scaling from modulators’ damage threshold. Second, laser characteristics can be actively regulated by adjusting pulsed pump source, indicating higher flexibility and controllability. In contrast to fast developed ~2 μm gain-switched fiber lasers^20^, the progress at ~3 μm waveband is slow. In 2001, B. C. Dickinson *et al*. demonstrated a 791 nm pumped gain-switched Er^3+^/Pr^3+^-codoped ZBLAN fiber laser at 2.7 μm with a pulse energy of 1.9 mJ. But only relaxation oscillations were achieved due to the use of an unideal flashlamp-pumped Ti:sapphire laser as the pump^21^. In 2011, M. Gorjan *et al*. presented stable gain-switching at 2.8 μm from a 976 nm diode pumped Er^3+^-doped ZBLAN fiber. The maximum average power of 2 W was obtained at a repetition rate of 100 kHz^22^. Recently, C. Wei *et al*. performed wavelength tuning (i.e., 2699 nm~2869.9 nm) of a gain-switched Er^3+^-doped ZBLAN fiber laser at a repetition rate of 20 kHz^23^. Very recently, Y. Shen *et al*. also demonstrated a wavelength tunable gain-switched Er^3+^-doped ZBLAN fiber laser at the range of 2710~2830 nm in which gain-switched mode-locking was observed for the first time^24^. However, until now, all the mid-infrared gain-switched fiber lasers emitted at a single waveband (~3 μm or ~2 μm).

In this report, we demonstrate a dual-waveband gain-switched Ho^3+^-doped ZBLAN fiber laser at ~3 μm and ~2 μm based on the mechanism of cascaded gain-switching for the first time. Stable ~3 μm gain-switched pulses at a repetition rate of 80 kHz and ~2 μm gain-switched pulses at a switchable repetition rate between 40 kHz and 80 kHz were produced simultaneously with a time delay of several μs. The proposed pulse-switching method could be also applied in multi-waveband pulses generation.

## Results

**Figure 1:**
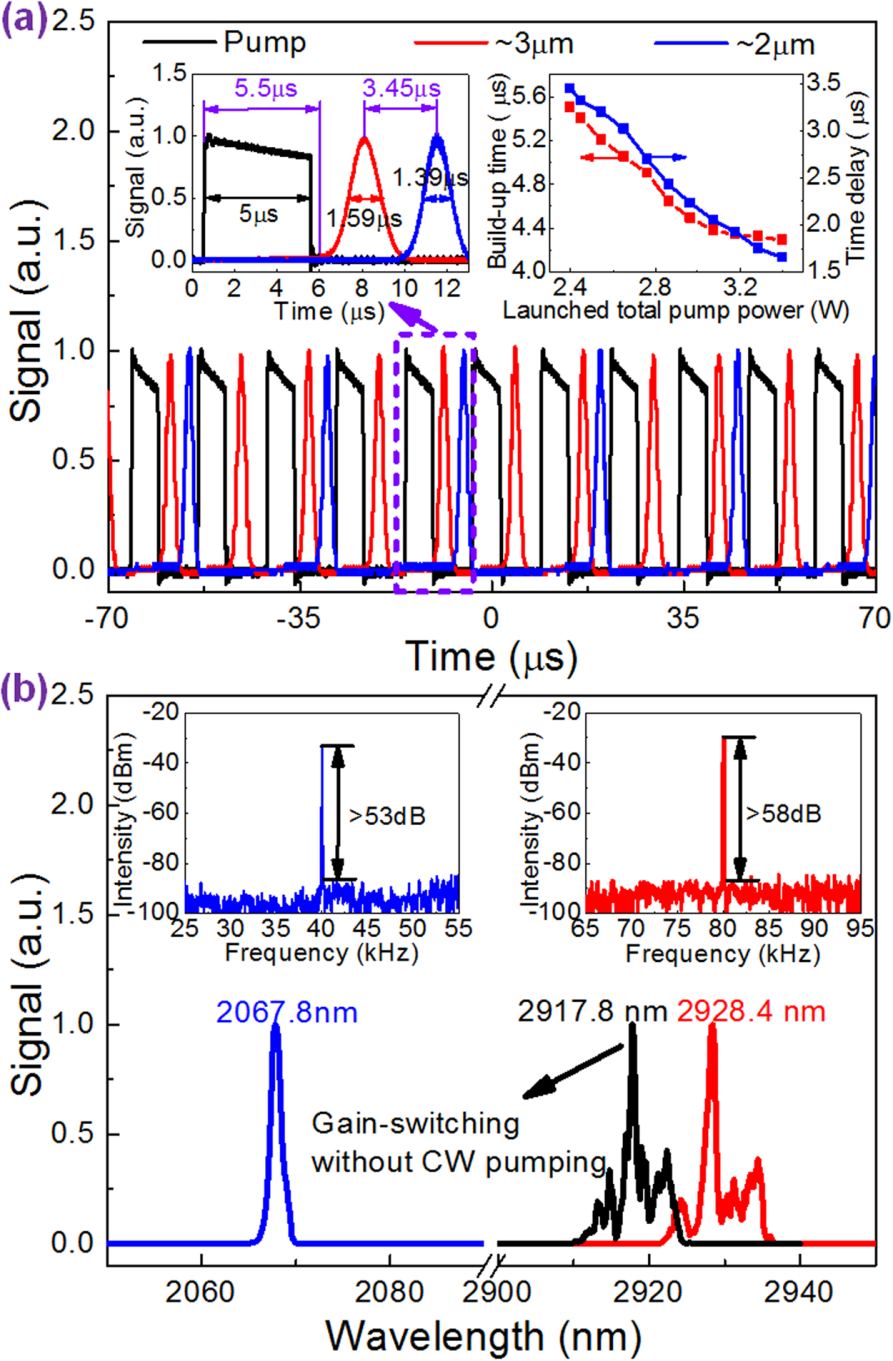
The output characteristics of “2-1” state. (**a**) Pulse trains and single pulse waveforms (left inset) at the LTPP of 2.4 W (LCWPP: 1.94 W, LPPP: 0.46 W), and build-up time and time delay versus the LTPP (right inset); (**b**) Optical and RF (insets) spectra at the LTPP of 3.4 W (LCWPP: 1.94 W, LPPP: 1.46 W). Note: the black optical spectrum was recorded at the LPPP of 1.8 W when switching off CW pump scheme.

In our experiment, the CW pump scheme was first switched on. Its launched pump power was adjusted to and clamped at 1.94 W, the threshold of ~2 μm laser. At this pump power, 92.6 mW ~3 μm CW laser was gotten. If further increasing the CW pump power, obvious ~3 μm self-pulsing was observed, its cause was also under investigation. Then the pulsed pump scheme was switched on, working at the available maximum modulation frequency of 80 kHz (limited by the large capacitance of the LDs) and a moderate duty cycle of 40% (corresponding to the pulse duration of 5 μs). The ~3 μm CW laser was immediately converted into unstable multi-pulsing which may be resulted from the combined effects of self-pulsing and gain-switching under low pulse pump power^25^. While the induced ~2 μm pulsing was also seen with sharply fluctuated amplitudes and varied pulse-to-pulse temporal intervals. Continue to increase the launched pulse pump power (LPPP) until 0.45 W, ~3 μm and ~2 μm pulsing became stronger and stronger but still unstable. When the LPPP was increased to 0.46 W at the fixed launched CW pump power (LCWPP) of 1.94 W. Stable ~3 μm gain-switched pulses at the same repetition rate of 80 kHz as pump pulses were obtained with a μs-order build-up time (calculated from front edge of pump pulse to that of ~3 μm pulse in a period). At this pump power, self-pulsing had been strongly suppressed by the gain-switching and almost negligible influence on the final output. With populations on ^5^I_7_ level being periodically modulated by ~3 μm gain-switched pulses, stable ~2 μm gain-switched pulses were then induced with a μs-order time delay (calculated from ~2 μm pulse peak to its adjacent ~3 μm pulse peak) but at a halved repetition rate of 40 kHz as shown in Fig. 1(a). This process was termed as cascaded gain-switching. In this case, to induce one ~2 μm gain-switched pulse, two ~3 μm gain-switched pulses were needed due to its low pulse energy. Thus this temporal state was called as “two for one” (“2-1”) state for short by us. The left inset of Fig. 1(a) shows the zoomed pump, ~3 μm and ~2 μm gain-switched pulses. Their pulse durations were 5 μs, 1.59 μs and 1.39 μs, respectively. The corresponding build-up time and time delay were 5.5 μs and 3.45 μs, respectively. This stable “2-1” state could be kept until the LPPP of 1.46 W at the fixed LCWPP of 1.94 W. Across this pump range, the build-up time and time delay decreased from 5.5 μs to 4.29 μs and 3.45 μs to 1.65 μs, respectively, with the increase of LPPP, as shown in the right inset of Fig. 1(a), as a result of faster populations accumulation on ^5^I_6_ and ^5^I_7_ levels. Figure 1(b) shows the optical and RF spectra of ~3 μm and ~2 μm gain-switched pulses measured at the LPPP of 1.46 W while LCWPP of 1.94 W. It is seen that the ~2 μm optical spectrum centered at 2067.8 nm is smooth but many peaks are involved in the ~3 μm optical spectrum centered at 2928.4 nm. To make this clear, the optical spectrum of stable gain-switched pulses only under pulse pumping (LPPP: 1.8 W) was also measured as shown in Fig. 1(b). The similar optical spectrum shape indicated the multi-peaks resulted from intra-cavity mode distribution instead of CW pumping. High SNRs of >58 dB (~3 μm) and >53 dB (~2 μm) also confirmed quite pure dual-waveband gain-switching.

**Figure 2:**
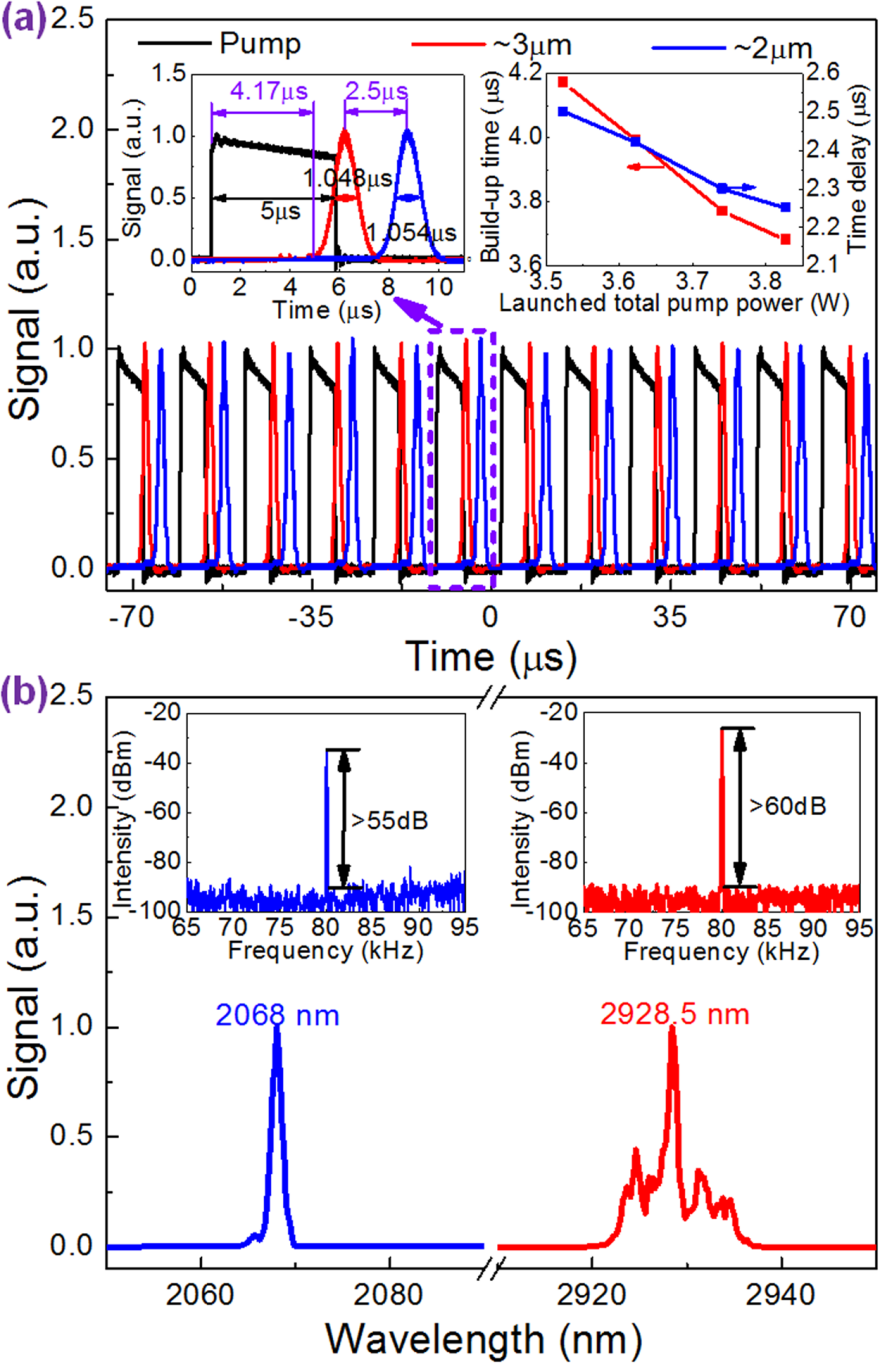
The output characteristics of “1-1” state. (**a**) Pulse trains and single pulse waveforms (left inset) at the LTPP of 3.52 W (LCWPP: 1.94 W, LPPP: 1.58 W), and build-up time and time delay versus the LTPP (right inset); (**b**) Optical and RF (insets) spectra at the LTPP of 3.83 W (LCWPP: 2.03 W, LPPP: 1.8 W).

Further increasing the LPPP to 1.58 W at the fixed LCWPP of 1.94 W, stable ~2 μm gain-switched pulses at the same repetition rate of 80 kHz as ~3 μm gain-switched pulses were gained instead as shown in Fig. 2(a). In this case, only one ~3 μm gain-switched pulse could induce one ~2 μm gain-switched pulse suggesting more populations accumulated on ^5^I_7_ level in one pump period as a result of the boosted ~3 μm pulse energy. This temporal state was thus called as “one for one” (“1-1”) state for short by us. The left inset of Fig. 2(a) shows the zoomed pump, ~3 μm and ~2 μm gain-switched pulses in one period, yielding the pulse durations of 5 μs, 1.048 μs and 1.054 μs, respectively. The corresponding build-up time and time delay were 4.17 μs and 2.5 μs, respectively. Note that at the LPPP range from 1.46 W to 1.58 W while the LCWPP of 1.94 W, it is the transition state jumping between “2-1” and “1-1” states. Until the allowable maximum LPPP of 1.8 W at the fixed LCWPP of 1.94 W, this stable “1-1” state could be always observed. At this moment, if further increasing the LCWPP at the LPPP of 1.8 W, this state was still maintained until the LCWPP of 2.03 W. The right inset of Fig. 2(a) displays the build-up time and time delay of stable “1-1” state with the increased launched total pump power (LTPP) from 3.52 W (LCWPP: 1.94 W, LPPP: 1.58 W) to 3.83 W (LCWPP: 2.03 W, LPPP: 1.8 W) as the process above. It is observed that both decreased almost linearly from 4.17 μs to 3.68 μs and 2.5 μs to 2.25 μs, respectively. At the LTPP of 3.83 W (LCWPP: 2.03 W, LPPP: 1.8 W), the optical and RF spectra were also measured as shown in Fig. 2(b). The center wavelengths were almost unchanged at 2068 nm and 2928.5 nm. And the SNRs were further improved to > 60 dB (~3 μm) and > 55 dB (~2 μm). Once the LCWPP was increased beyond 2.03 W at the maximum LPPP of 1.8 W, however, unstable ~3 μm multi-pulsing was observed due to excessive populations accumulated on ^5^I_6_ level which cannot be fully depleted by one ~3 μm gain-switched pulse and the influence of self-pulsing mechanism, then inducing unstable ~2 μm gain-switching. Note that the way to adjust both CW and pulsed pump schemes wouldn’t influence the laser characteristics. It meant that both temporal state and output performance of dual-waveband gain-switched pulses were certain at the specific pair of LCWPP and LPPP.

**Figure 3:**
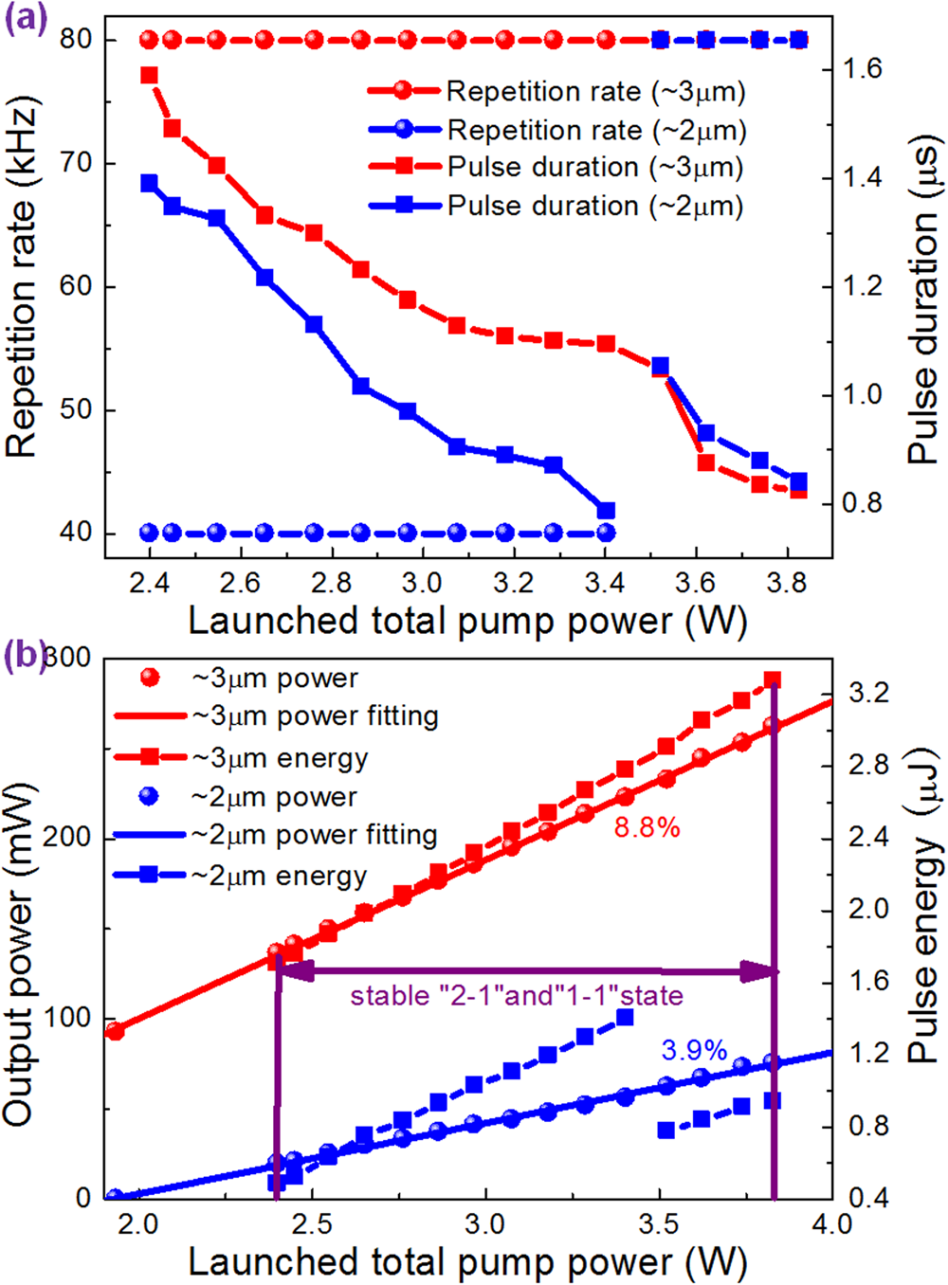
The output performances of dual-waveband gain-switched fiber laser. (**a**) Repetition rate and pulse duration and (**b**) output power and pulse energy as a function of the LTPP.

Figure 3(a) shows the repetition rates and pulse durations of both ~3 μm and ~2 μm gain-switched pulses as a function of the LTPP. It is seen that ~3 μm gain-switched pulses always operate at the same repetition rate of 80 kHz as pump pulses. But ~2 μm gain-switched pulses presented a suddenly doubled repetition rate from 40 kHz to 80 kHz with increasing the LTPP from 3.4 W (LCWPP: 1.94 W, LPPP: 1.46 W) to 3.52 W (LCWPP: 1.94 W, LPPP: 1.58 W). While the ~3 μm gain-switched pulse duration decreased monotonically from 1.59 μs to 0.824 μs with increasing the LTPP from 2.4 W (LCWPP: 1.94 W, LPPP: 0.46 W) to 3.74 W (LCWPP: 1.94 W, LPPP: 1.8 W) then to 3.83 W (LCWPP: 2.03 W, LPPP: 1.8 W). The ~2 μm gain-switched pulse duration decreased from 1.39 μs to 0.787 μs with the LTPP increasing from 2.4 W (LCWPP: 1.94 W, LPPP: 0.46 W) to 3.4 W (LCWPP: 1.94 W, LPPP: 1.46 W). However, it had a sudden increase from 0.787 μs to 1.054 μs with the increased LTPP from 3.4 W (LCWPP: 1.94 W, LPPP: 1.46 W) to 3.52 W (LCWPP: 1.94 W, LPPP: 1.58 W). It was due to the reduced populations accumulated on ^5^I_7_ level used for forming one ~2 μm gain-switched pulse since populations supplier became one ~3 μm gain-switched pulse from two. Further increasing the LTPP from 3.52 W (LCWPP: 1.94 W, LPPP: 1.58 W) to 3.74 W (LCWPP: 1.94 W, LPPP: 1.8 W) then to 3.83 W (LCWPP: 2.03 W, LPPP: 1.8 W), the ~2 μm gain-switched pulse duration continued to decrease from 1.054 μs to 0.84 μs. Their output powers and pulse energies as a function of the LTPP were also recorded and calculated as show in Fig. 3(b). Both their output powers increased almost linearly with the increased LTPP. At the LTPP of 3.83 W (LCWPP: 2.03 W, LPPP: 1.8 W), the maximum output power of 262.14 mW of ~3 μm gain-switched pulses at a slope efficiency of 8.8% was achieved. The corresponding maximum pulse energy was 3.28 μJ. While the maximum output power of 75.23 mW of ~2 μm gain-switched pulses was also obtained at a slope efficiency of 3.9%. But their maximum pulse energy of 1.4 μJ was gained at the LTPP of 3.4 W (LCWPP: 1.94 W, LPPP: 1.46 W) as a result of comparatively low repetition rate of 40 kHz. At this pump power, the long-term stabilities of output powers were also measured. The low power fluctuations of ±4% (~3 μm) and ±3% (~2 μm) within 5 hours indicated the high stability of this dual-waveband pulsed laser. As addressed before, once the LCWPP exceeded 2.03 W at the allowable maximum LPPP of 1.8 W, the unstable ~3 μm multi-pulsing appeared leading to unstable ~2 μm gain-switching, thus impeded further output scaling of stable dual-waveband gain-switching. At this time, we adjusted the LPPP slightly beyond 1.8 W but at a very short time, and observed the reappearance of stable dual-waveband gain-switching. Although no more data were recorded in order to keep LDs damage from overloaded operation beyond the maximum allowable LPPP of 1.8 W, the phenomenon indicated further output scaling was indeed limited by the LPPP in our case. Actually, the similar phenomenon had been observed in a previously established hybrid pumped ~2 μm gain-switched Tm^3+^-doped fiber laser^25^ which manifested there was always an allowable maximum LPPP for each specific LCWPP to realize stable gain-switching. Besides, fast gain-switching using narrow pulse pumping was verified to be a feasible way to postpone the arrival of multi-pulsing^26^. The μs-order pump pulse duration in our case was thus another limitation factor for output scaling.

Moreover, the effects of pump modulation frequency and pulse duration on the temporal evolution of dual-waveband laser were also experimentally studied. First, the pump pulse duration was fixed at 5 μs by adjusting the cycle duty when the modulation frequency was varied. We found that at a little lower modulation frequency of 70 kHz, the similar temporal evolution was observed just with lower maximum power and energy. However, when the modulation frequency was decreased to 60 kHz and even lower, it became hard to observe stable “1-1” or even “2-1” state as the way above to increase LTPP due to the only multi-pulsing of ~3 μm laser as a result of limited PLLL. Second, the pump modulation frequency was fixed at 80 kHz. At the available lowest cycle duty of 20% which depended on the used function generator of our modulator, although stable “1-1” state was not observed mainly due to the limited PLLL, it was reasonably predicted. However, when the cycle duty was increased to 50%, multi-pulsing of ~3 μm laser instead of stable “1-1” state followed stable “2-1” state due to the reduced pump pulse energy threshold of ~3 μm multi-pulsing with the increased cycle duty. This point has been verified in our recent another investigation on ~3 μm gain-switched Ho^3+^-doped ZBLAN fiber laser. If continuing to increase the cycle duty to 60% and even larger, both “2-1” and “1-1” states could not be observed any more. The results indicated that high modulation frequency and small pump pulse duration facilitated stable dual-waveband gain-switched pulses generation.

## Discussion

For gain-switching, pulse duration was a key parameter associated with practical applications. In this case, the shortest pulse durations of ~3 µm and ~2 µm gain-switched pulses were 824 ns and 787 ns, respectively which were much larger than some obtained in the wavelength region of 1~2 µm (as small as several hundreds and even tens of ns)^20^. According to our recent investigation on ~3 µm single-waveband gain-switching (unpublished) combined with the previously reported results^20,27^, it was found that the gain-switched pulse duration was related to a set of parameters mainly including pump pulse energy, pump modulation frequency, pump pulse duration, cavity length, output coupler ratio and operation wavelength. For the former three which were relatively feasible to control for our current scheme, higher pump pulse energy, lower pump modulation frequency and smaller pump pulse duration were beneficial for obtaining narrower pulses. In our case, however, the modulation frequency could not be tuned to too low in order to get stable dual-waveband gain-switching. Thus, the allowable maximum pump pulse energy and minimum pump pulse duration limited the achievement of shorter pulse duration. For the latter two, although there were optimal output coupler ratio and operation wavelength to minimize pulse duration, additional components (e.g., partially reflected mirror, wavelength selector) were needed which significantly complicated the system, despite the compact but not cost-effective mid-infrared ZBLAN FBG^28^. For the cavity length (=gain fiber length + free-space length) directly related to the pulse duration, the intra-cavity free-space length in our scheme was about 15 cm which was hard to be further shortened considering the self-volume of the mount used for the mirror/lens. On the other hand, it was impractical to sharply shorten the gain fiber as well in order to maintain enough gain since it was lowly doped.

## Conclusions

In summary, we demonstrated ~3 μm and ~2 μm dual-waveband pulses from a cascaded gain-switched Ho^3+^-doped ZBLAN fiber laser in a compact linear cavity. CW and pulse lasers at 1150 nm were used to pump the scheme simultaneously where the CW laser aimed at providing populations for 5I6 level while the pulse laser acted as a switch to trigger the ~3 μm pulses. At the pump modulation frequency of 80 kHz and cycle duty of 40%. Stable ~3 μm gain-switched pulses at the same repetition rate of 80 kHz were first obtained. With a μs-order time delay, stable ~2 μm gain-switched pulses were also achieved at a switchable repetition rate between 40 kHz and 80 kHz. The shortest pulse durations of ~3 μm and ~2 μm gain-switched pulses were 0.824 μs and 0.787 μs, respectively. The maximum pulse output power of 262.14 mW (~3 μm) and 75.23 mW (~2 μm) were mainly limited by 1150 nm pulse pump. In the next step, further output scaling of dual-waveband gain-switched pulses would be carried out by introducing more powerful and narrower 1150 nm pulse pump sources (e.g., pulsed version of 1150 nm Yb^3+^-doped silica fiber laser^29^) combined with cascaded Ho^3+^-doped ZBLAN fiber based MOPA scheme considering their high SNRs. This demonstration puts forward a universal and compact approach to yield dual- and even multi-waveband pulses, and is beneficial for some applications in the mid-infrared region.

## Methods

### Design of gain-switched fiber laser

**Figure 4:**
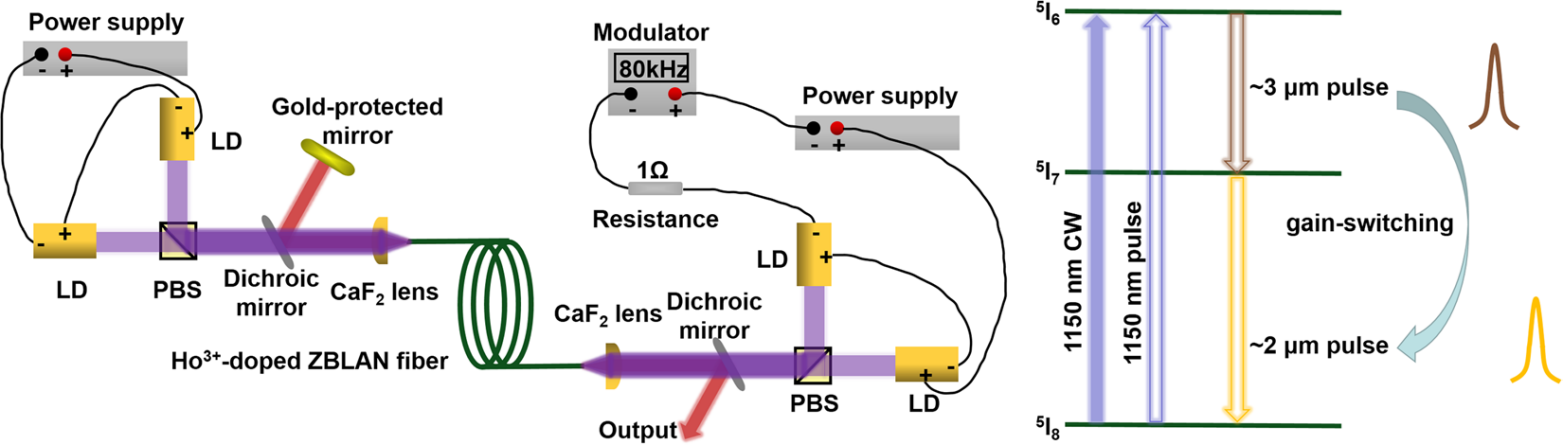
Schematic of the dual-waveband gain-switched Ho^3+^-doped ZBLAN fiber laser. PBS is polarizing beam splitter. Inset: schematic of energy level structure of Ho^3+^-doped ZBLAN fiber and the mechanism of cascaded gain-switching under CW and pulse pumping simultaneously.

The schematic of the laser is shown in Fig. 4. The gain fiber was a custom-designed double-clad Ho^3+^-doped ZBLAN fiber (FiberLabs, Japan). It has a D-shaped inner clad with a diameter of 125 μm across the circular cross section and a NA of 0.5. Its core diameter and NA are 10 μm and 0.16, respectively. To obtain ~3 μm and ~2 μm components easier simultaneously (the simplified energy level structure is shown in the right inset of Fig. 4), low Ho^3+^ ions dopant concentration of 1.2 mol.% was selected to weaken energy transfer between Ho^3+^ ions thus reducing ~2 μm laser threshold. Its clad absorption coefficient at 1150 nm was measured to be 0.28 cm^−1^ using the cutback method and length was 4.5 m. Based on our off-the-shelf commercial 1150 nm LDs (Eagleyard Photonics Berlin) in lab, double-side pumped arrangement was designed to provide enough pumping for dual-waveband emissions. However, it would bring us the trouble that the pump pulses from two ends would temporally asynchronously change local gain in the cavity hence leading to chaotic gain-switching. To circumvent this problem, CW pumping was meanwhile introduced just to provide populations for ^5^I_6_ laser upper level when pulse pumping acted as a switch to trigger the ~3 μm and then ~2 μm pulses. The CW pump scheme includes two polarized combined 1150 nm LDs using a polarizing beam splitter (PBS). The output CW laser was launched into the gain fiber after focusing using a plano-convex CaF_2_ lens (Thorlabs, LA5315) at a launching efficiency of 82%. A dichroic mirror with a transmission of 94% at 1150 nm and a high reflection of >95% at ~3 μm and ~2 μm was positioned between the PBS and CaF_2_ lens at an angle of ~30% with respect to the pump beam. It was used to steer the ~3 μm and ~2 μm laser from the fiber core onto a gold-protected mirror as one side of the cavity. To prevent intra-cavity parasitic lasing, the fiber end close to the CW pump scheme was cleaved at an angle of 8°. The pulsed pump scheme is similar to the CW one just with additionally inserting a home-made modulator and 1 Ω resistance (monitoring instantaneous current) into the loop. The output pulsed laser was launched into the gain fiber using a same scheme as before. Here the fiber end was perpendicularly cleaved as the other side of the cavity and output coupler with the help of 4% Fresnel reflection. Another dichroic mirror same as before was also positioned between the PBS and CaF_2_ lens to steer the ~3 μm and ~2 μm pulses out from the system.

### Measurement of laser output characteristics

The dual-waveband pulses were distinguished using a dichroic mirror (high transmission/reflection at ~3/2 μm) and then detected using an InAs detector (Judson, J12-18C-R01M) and an InGaAs (EOT ET-5000F), respectively, both connected with a 500 MHz digital oscilloscope. The radio frequency (RF) spectrum was captured by a RF spectrum analyzer (YIAI, China, AV4033A, 30Hz–18GHz). A monochromator based on nitrogen cooled photodiode (Princeton instrument Acton SP2300) was used to measure optical spectrum.
